# Application and Progress of Raman Spectroscopy in Male Reproductive System

**DOI:** 10.3389/fcell.2021.823546

**Published:** 2022-01-12

**Authors:** Feng Zhang, Yiling Tan, Jinli Ding, Dishuang Cao, Yanan Gong, Yan Zhang, Jing Yang, Tailang Yin

**Affiliations:** ^1^ Reproductive Medical Center, Renmin Hospital of Wuhan University, Wuhan, China; ^2^ College of Optometry and Ophthalmology, Tianjin Medical University, Tianjin, China; ^3^ Department of Clinical Laboratory, Renmin Hospital of Wuhan University, Wuhan, China

**Keywords:** Raman spectroscopy, male reproductive system, sperm, prostate, diagnosis, application

## Abstract

Raman spectroscopy is a fast-developing, unmarked, non-invasive, non-destructive technique which allows for real-time scanning and sampling of biological samples *in situ*, reflecting the subtle biochemical composition alterations of tissues and cells through the variations of spectra. It has great potential to identify pathological tissue and provide intraoperative assistance in clinic. Raman spectroscopy has made many exciting achievements in the study of male reproductive system. In this review, we summarized literatures about the application and progress of Raman spectroscopy in male reproductive system from PubMed and Ovid databases, using MeSH terms associated to Raman spectroscopy, prostate, testis, seminal plasma and sperm. The existing challenges and development opportunities were also discussed and prospected.

## 1 Introduction

**FIGURE 1:**
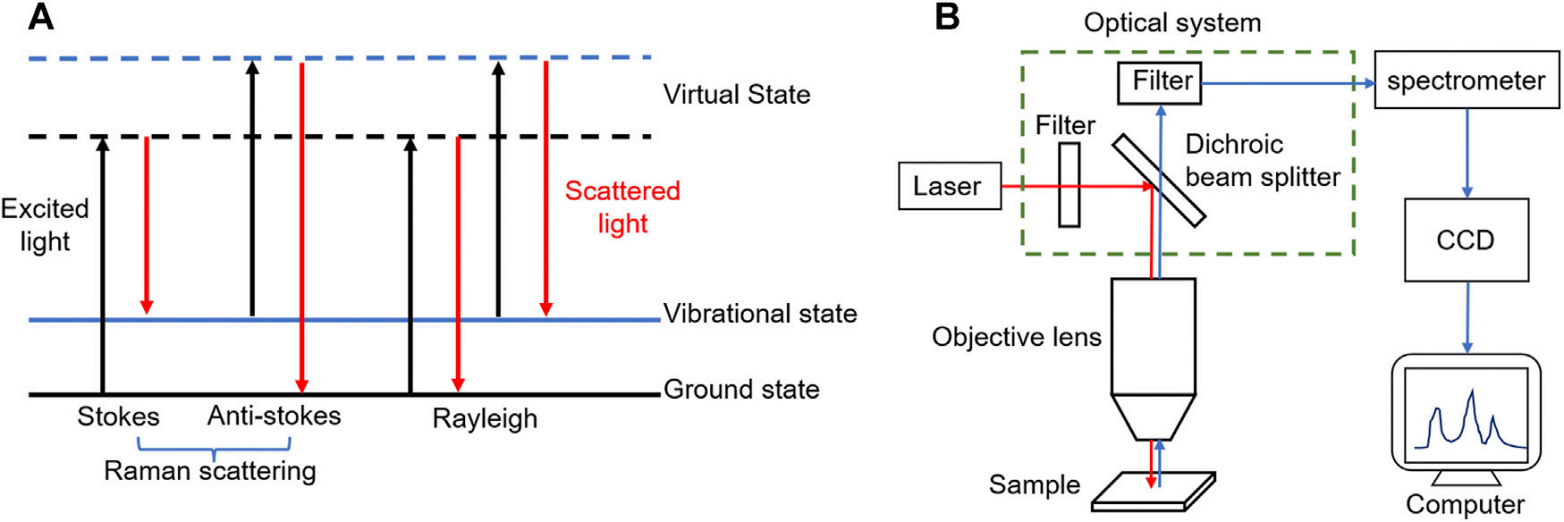
**(A)** After the electron absorbs the excited light energy (black line), the energy level transition occurs, from the ground state or vibrational state to the virtual state, and then releases the energy in the form of scattered light (red line). When the release energy is equal to the absorption energy, the electron energy level remains unchanged (Rayleigh); when the release energy is less than the absorption energy, the overall energy level of the electron increases (Stokes); on the contrary, the energy level decreases (Anti-stokes). **(B)** The schematic diagram of a typical Raman spectrometer, including excitation light source, optical system, spectrometer, CCD detector and computer processing system.

Raman scattering is a kind of inelastic scattering effect of molecules to photons, the frequency of scattered light is different from that of excited light, which was discovered by Indian physicist C.V. Raman in 1928 (Raman and Krishnan, 1928). Raman scattering is divided into two types: when the frequency of scattered light is less than the frequency of excited light, it is called Stokes Raman scattering; if the frequency of scattered light is greater than that of excited light, it is called anti-Stokes Raman scattering. Different from Raman scattering, Rayleigh scattering is a kind of elastic scattering, which means the frequency of scattered and excited light are equal (Wang et al., 2018) (Figure 1A).

The Raman spectrum based on Raman scattering effect is analyzed to obtain the information of molecular vibrational energy level (lattice vibrational energy level) and rotational energy level structure, containing a lot of available information. The position and intensity of Raman shift, the shape of Raman peak and full width at half maximum (FWHM) are all important basis for the identification of chemical bonds, functional groups, crystallinity and strain (Bogliolo et al., 2020). Raman spectrometer is an instrument for measuring Raman spectral signals, usually composed of five parts: excitation light source, optical system (including filter, dichroic beam splitter, etc.), spectrometer, detector (i.e. CCD detector) and computer processing system (Mallidis et al., 2014; Cordero et al., 2018; Wang et al., 2018) (Figure 1B). The common confocal micro-Raman spectrometer used commonly in the market is the coupling of Raman spectrometer and optical microscope, which can greatly improve the horizontal and vertical spatial resolution to realize the analysis of micron-scale samples (Meister et al., 2010; Huang et al., 2014; Gomes da Costa et al., 2019).

Raman spectroscopy has the advantages of rich information, simple sample preparation, little interference with water, non-labeling, non-invasive, non-destructive and accurate discrimination, so it has been widely popular in chemistry, materials, physics, polymer, biomedicine and other fields (Cardinal et al., 2017; DePaoli et al., 2020). However, the Raman scattering intensity is very weak, only about 10^–7^ of the excited light intensity (Du et al., 2021). In order to overcome the shortcomings of conventional Raman spectroscopy, such as low inherent sensitivity, strong fluorescence interference and limited application, a series of Raman spectral derivation techniques have been developed, including surface enhanced Raman scattering (SERS), coherent anti-Stokes Raman spectroscopy (CARS), stimulated Raman spectroscopy (SRS), spatial offset Raman spectroscopy (SORS), resonance Raman spectroscopy (RRS), etc. (Brusatori et al., 2017; Nicolson et al., 2021; Ramya et al., 2021), which makes it has a wider application scene and play unique advantages and key roles in specific researches.

At present, Raman spectroscopy and its derivative techniques have been applied to biomedical researches *in vitro* and *in vivo*. Pathological diagnosis of tumors is the most common *in vitro* study, such as oral cancer (Sahu and Krishna, 2017), skin cancer (Zhao et al., 2017), bladder cancer (Liu et al., 2021), renal cell carcinoma (Jin et al., 2020), brain cancer and breast cancer (Abramczyk et al., 2020). What is exciting is that many studies have been clinically transformed to determine the surgical margins of tumors and intraoperative assistance (Santos et al., 2017; Hubbard et al., 2019; DePaoli et al., 2020). In addition, many *in vitro* studies on cells and body fluids have been reported (Durrant et al., 2019; Wallace and Masson, 2020). Thanks to the development of portable Raman spectrometer, it has become a new research hotspot to combine it with endoscopes such as gastroscope (Wang et al., 2015) and enteroscope (Lane et al., 2018). Raman probe was sent to the focus with the help of endoscope, and signals were excited and collected through a handheld optical fiber device, so as to realized real-time visual *in vivo* detection.

**FIGURE 2:**
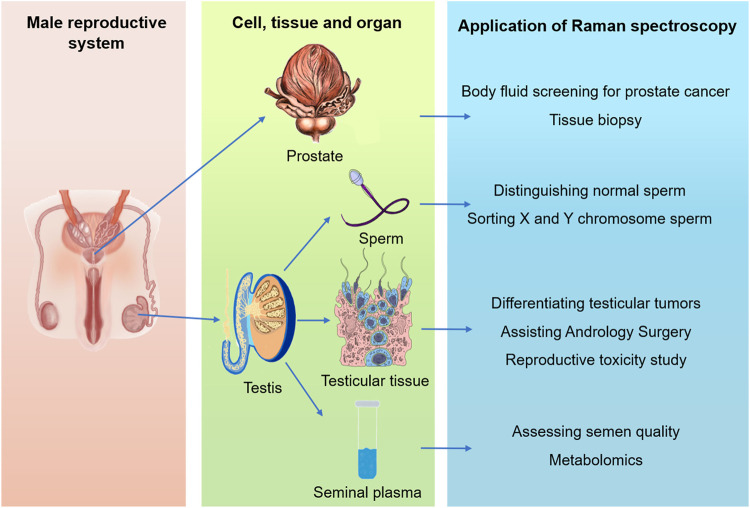
Summary of the applications of Raman spectroscopy in male reproductive system, including organ (prostate and testis), tissue, cell and body fluid levels.

In this review, we gathered literatures about the application of Raman spectroscopy in andrology and other medical disciplines from PubMed and Ovid databases, using MeSH terms associated with Raman spectroscopy, prostate, testis, seminal plasma and sperm, and summarized the application progress of Raman spectroscopy and its derivative techniques in male reproductive system from the four dimensions of prostate, testis, seminal plasma and sperm (Figure 2). This review emphasized its clinical and scientific research applications in prostate and sperm analysis, and probed into the challenges and opportunities of Raman detection of single living spermatozoa. The application and development direction of Raman spectroscopy in male reproductive system were also discussed and prospected.

## 2 Application in Prostate Diseases

The biochemical changes of prostate tissue caused by genetic mutations take precedence over changes in morphology and structure. While these biochemical changes can be detected by Raman spectroscopy, and spectroscopic analysis allows visualization of the local microscopic distribution of the various components. By collecting and analyzing enough specific optical data of disease tissues, a unique “optical fingerprint” database of prostate diseases can be established, which has a positive impact on the screening, diagnosis and prognostic assessment of prostate diseases.

By comparing the intensity of characteristic peaks corresponding to glycogen and nucleic acids, Crow et al. (2003) had demonstrated that Raman spectroscopy could accurately identify benign prostate hyperplasia (BPH) and three different grades of prostate adenocarcinoma *in vitro*. In a follow-up study, for the purpose of distinguishing benign samples (BPH and prostatitis) from malignant samples (prostate cancer), Crow et al. (2005) constructed a prostate diagnosis algorithm with an accuracy of 86% in total. It was suggested that the clinical Raman system could provide an accurate and objective method for *in vitro* diagnosis of prostate cancer by which so many discoveries were founded.

### 2.1 Body Fluid Screening for Prostate Cancer

For the aim of analyzing and differentiating prostate cells *in vivo* by significant spectral peak differences at 1,297 cm^−1^ (lipids) and 1,580 cm^−1^ (proteins) from PC-3 (prostate) and MGH-U1 (bladder) cancer cells, Harvey et al. (2008) developed a Raman optical tweezer system, which was also observed when examining various formalin-fixed urothelial cells using optical tweezers (Harvey et al., 2009). These two peaks were also clearly prominent in the Raman spectroscopic analysis curves of prostate cancer pathological tissue obtained by Patel and Martin (2010) and were exactly valuable in determining the presence of malignant prostate cancer cells in urine and peripheral fluid.

While examining the Raman spectra of urine from normal subjects and prostate cancer patients, Del Mistro et al. (2015) made full use of principal component analysis (PCA) and linear discriminant analysis (LDA) to reduce dimension and differentiate the spectral data, and found that the intensity of spectra assigned to creatinine and blood uric acid purine was significantly different between the two groups, as a result, a diagnostic model was constructed based on the difference spectra. It was 100% sensitivity, 89% specificity and 95% accuracy that diagnostic model could achieve, which indicated that urine specimens could also potentially be used for prostate cancer screening. Owing to these discoveries so far, there is no doubt that detecting more accurately spectral peak differences, implementing more accurate algorithms and building more accurate diagnostic models have become a new challenge. With the help of Raman spectroscopy and convolutional neural network (CNN) algorithm to study urine samples, Chen et al. (2021) established a non-invasive diagnostic method for prostate carcinoma. Obviously, the results of urine Raman spectroscopy showed that the intensity of characteristic peaks of lipids, nucleic acids and some amino acids of prostate carcinoma and BPH could be distinguished, and then these data were used to train an intelligent diagnostic model of CNN algorithm to deep study, and the idea of using urine Raman spectroscopy combined with deep learning technology to diagnose prostate carcinoma provides a reference for the application of artificial intelligence in the field of clinical medical research.

Obtaining a total of 240 SERS spectra from the sera of 40 prostate carcinoma subjects and 40 BPH subjects, Chen et al. (2017) analyzed the spectral data of patients in the control, prostate carcinoma and BPH groups by taking advantage of multivariate statistical techniques including PCA and LDA diagnostic algorithms, which demonstrated that the use of SERS combined with PCA-LDA diagnostic algorithm to analyze serum was a promising diagnostic and clinical evaluation tool for prostate carcinoma. Stefancu et al. (2018) used PCA-LDA to combine SERS spectra of serum samples with serum PSA levels and found that the accuracy of differentiating prostate cancer from non-malignant lesions could be further improved. Li S. et al. (2014) used silver colloid to detect serum SERS spectra of prostate cancer patients and normal volunteers with PSA level in the gray region and obtained significant differences between normal and cancer serum SERS spectra, suggesting that label-free diagnosis of prostate cancer using serum SERS spectra of peripheral blood samples showed great potential in characterizing the differences in the biochemical composition of prostate carcinoma and BPH patients with PSA levels in the gray area. Combined with PCA-LDA multivariate analysis, this diagnostic technique could achieve high sensitivity, specificity and accuracy. They further developed three support vector machine (SVM) classifier models, such as linear kernel function, polynomial kernel function and RBF kernel function. They evaluated them with measured serum SERS profiles and found that the diagnostic accuracy of all three SVM diagnostic models were higher than that of the PCA-LDA algorithm. Among them, the RBF kernel SVM classifier model had a diagnostic accuracy of 98.8%. These results suggested that the diagnostic performance of SVM was superior to that of the PCA algorithm. This preliminary study indicated that the combination of the serum SERS technique and the SVM algorithm had great potential for noninvasive screening of prostate cancer by peripheral blood samples.

Shao et al. (2020) showed that the diagnostic sensitivity, specificity and accuracy of the PCA-LDA model based on Raman spectroscopy of prostatic fluid were 75, 75, and 75%, respectively, while the diagnostic sensitivity, specificity and accuracy of serum were 60, 76.5, and 68%. It can be seen obviously that both of them can distinguish well between prostate cancer and BPH, and prostate fluid has better diagnostic accuracy than serum.

Beyond all doubt, Raman spectroscopy demonstrated good accuracy in diagnosing prostate cancer on multiple biological fluids just like serum, urine, and prostate fluid. Although the sample sizes of the aforementioned studies were generally small, a large number of external population samples are required to validate these results, it provides a new idea for early *in vitro* low-cost screening of the prostate. Among them, the use of Raman spectroscopy to obtain the spectra of body fluids to construct a database to diagnose by manual simulation is becoming an unstoppable trend in the diagnosis of prostate disease (Pan et al., 2019).

### 2.2 Tissue Biopsy for Prostate Diseases

Fine-needle aspiration biopsy of the prostate tissue is considered as gold standard for the diagnosis of most prostate diseases, but there are still some missed and misdiagnosed rates, and the pathological grading of the puncture is often inconsistent with the postoperative pathological grading. In addition, all specimens require special handling and experienced pathologists to make the diagnosis, which is costly and time-consuming. Therefore, it is of great significance to explore new techniques and methods for cheap, rapid and accurate diagnosis of prostate diseases. Crow et al. (2003) found that Raman spectroscopy could effectively distinguish among normal prostate tissue, hyperplastic tissue and adenocarcinoma tissue by comparing the peak intensity of characteristic peaks corresponding to glycogen and nucleic acid. They further distinguished three pathological types of prostate adenocarcinoma with Gleason score <7, Gleason score = 7 and Gleason score >7 by constructing an algorithm. In a study by Stone et al. (2007), prostate cancer cells were found to have a higher nuclear/cytoplasmic ratio and higher DNA, choline and glyceric acid concentrations than prostate hyperplastic tissue. Based on the differences in the biochemical composition like proteins, lipids, nucleic acids and amino acids, and corresponding Raman bands in normal, BPH and prostatic adenocarcinoma, Lopes et al. (2011) developed a diagnostic algorithm combined PCA with Mahalanobis distance discriminant analysis to identify normal, BPH and adenocarcinoma tissues from *in vitro* prostate biopsy fragments and then developed a spectral diagnostic model for tissue classification. Prostate lesion samples were classified into three histological groups in this model with 100% sensitivity and specificity. And the Raman spectroscopy biochemical model of large samples showed that from normal to BPH and adenocarcinoma samples, the relative content of collagen decreased with the severity of the disease, while the content of cholesterol, fat cells and smooth cells increased. The number of smooth cells and collagen could be used to identify disease states with high sensitivity and specificity. Silveira et al. (2014) established Euclidean distance discrimination model by relative concentrations of phosphatidylcholine and water which had a sensitivity of 75% and specificity of 74% for prostate carcinoma diagnosis, and had a sensitivity of 69% and specificity of 89% for Gleason score >7 group, both of which opened the way for the application of Raman spectroscopy in the *in vivo* clinical setting. Artemyev et al. (2021) constructed analytical discriminative models of Raman spectra of prostate tissue using the projection to latent structure data analysis (PLS-DA) method, irradiating prostate tissue with different wavelengths such as 532 and 785 nm. These models could divide the Raman spectra of prostate carcinoma and the BPH into validation datasets and could detect biochemical alterations in the diseased tissue with high accuracy within a certain range.

Raman spectroscopy has been believed to have the potential to guide the determination of tumor margins for prostate radical surgery. Aubertin et al. (2018a) used a handheld Raman device to perform Raman spectroscopy on fresh prostate tissue from 32 patients after puncture and found it competent to differentiate between benign and malignant prostate with AUC (Area under curve, Roc curve) value at 0.93, and the AUC values for determining prostate tumor grading were 0.83–0.95, indicating that Raman spectroscopy could also effectively detect and analyze fresh prostate puncture specimens and might improve the surgical resection rate at radical operation. Thus, Raman spectroscopy could be used as a complement to intraoperative histopathological analysis. As a result of Raman probe suitable for endoscopic and laparoscopic or open surgery, the study work paved the way for *in vivo* studies.

## 3 Application in Testis Study

### 3.1 Identification of Testicular Tumors

It is the identification of pathological tissues of tumors that the application of Raman spectroscopy in clinical medicine still focuses on nowadays. Owing to the low incidence of testicular tumors presumably, the research progress of Raman spectroscopy in testicular tumors is astonishingly slow. Originating in the study of testicular microlithia samples, the first report demonstrated effectively that glycogen surrounded by microlithiasis located in the seminiferous tubules was associated with the occurrence of germ cell tumors (De Jong et al., 2004). While its homogeneity and its representativeness in seminoma are still controversial, TCam-2 cells has been the main *in vitro* model for the study of seminoma. Eppelmann et al. (2013) discovered that the Raman spectrum of TCam-2s consists of three different spectral modes. What’s more, PCA, cluster analysis and local spectral angle analysis further demonstrated that TCam-2s contained two different types of cells. One of them included two subgroups, and undoubtedly there were so many differences in protein expression, which further confirmed the existence and generational differences of different subtypes of cells at the same time. It was the unique ability of Raman spectroscopy to detect the heterogeneity of TCam-2s that this study verified, which also initially demonstrated the possibility of Raman spectroscopy to identify testicular tumor cells by detecting cell biochemical characteristics.

### 3.2 Assisting Andrology Surgery

Raman spectroscopy has been regarded as an auxiliary diagnostic method in surgical operations in many studies recently, which also has a very good application prospect in fine surgery (Aubertin et al., 2018b; Hubbard et al., 2019; DePaoli et al., 2020). Taking the lead in constructing a Sertoli-only syndrome rat model, Osterberg et al. (2014) demonstrated that the signal intensity of the seminiferous tubules with spermatogenesis and the sertoli-only tubules at 1,000 cm^−1^ and 1,690 cm^−1^ were significantly different. Under the identification of PCA, the sensitivity and specificity were 96 and 100% respectively. The area under the receiver operating characteristic curve (ROC) of tubules with spermatogenesis predicted by Raman spectroscopy was 0.98, which proved forcefully that Raman spectroscopy could identify accurately seminiferous tubules with spermatogenesis. In the same year, using human testicular tissue as the material, another study verified the clinical application potential of this technology to identify seminiferous tubules with spermatogenic function (Liu and He, 2014). Compared with obstructive azoospermia (OA) patients who had normal spermatogenesis, non-obstructive azoospermia (NOA) patients without spermatogenesis had higher spectral intensity of certain peaks in the seminiferous tubules, such as 1,001 cm^−1^, 1,152 cm^−1^, 1,515 cm^−1^ and 1,658 cm^−1^ assigned to protein. Consistent to Raman spectra, histochemical results also demonstrated that NOA tubules had a higher protein content. Perhaps owing to the increase in extracellular matrix (ECM) and collagen deposition, it resulted in the thickening of the lamina propria of the testis. The Raman spectroscopy technique for distinguishing NOA and OA testicular tissue samples based on the spectral difference had a sensitivity and specificity of 90 and 85.71% respectively, which indicated that Raman spectroscopy could be applied as a new and potentially useful tool to guide surgeons in microscopy testicular sperm extraction (micro-TESE) to improve sperm retrieval.

### 3.3 Study on Reproductive Toxicity of External Adverse Factors

On account of the unique anatomical location and the fact that sperm cannot repair genetic material like other tissue cells, the testis is very sensitive to external unfavorable factors, including endocrine disruptors, heavy metals, electromagnetic radiation and other physical and chemical factors. Raman spectroscopy and Fourier transform infrared spectroscopy (FTIR) can both identify the chemical bonds and functional groups based on the vibrational frequencies and associated energy levels in molecules. However, the former is based on the change of polarizability and is more suitable for detecting non-polar bonds, while the latter is induced by the change of dipole moment and is more competent for polar bonds. FTIR and Raman spectroscopy have their own advantages, so they are often used together to complement each other (Wang et al., 2018; Hosu et al., 2019). Combining Raman spectroscopy, attenuated total reflection Fourier transform infrared spectroscopy (ATR-FTIR) and multivariate analysis, Duan et al. (2019) identified the organisms molecular changes of testicular tissues and cells treated with the estrogen endocrine disruptor 4-nonylphenol which herald the possibility of Raman spectroscopy in the study of testicular toxicology. Immediately afterwards, another study reported the use of Raman spectroscopy to determine the reproductive toxicity of testis caused by cadmium, suggesting that the Raman peak changes at 746 cm^−1^, 996 cm^−1^, 1,086 cm^−1^, 1,244 cm^−1^, and 1,587 cm^−1^ were attributed to the decrease of mitochondrial cytochrome c and the changes of ribose and nucleic acid after cadmium treatment, which indicated the decrease of cell mitochondrial content and the increase of genomic instability after cadmium treatment (Liu et al., 2020). People living in modern society often only rejoice in the convenience electromagnetic waves bring to life, without thinking about whether the invisible electromagnetic field will push an invisible threat to human health. Koziorowska et al. (2020) conducted an *in vitro* study on the effects of ultra-low frequency electromagnetic fields on the testicular tissue of roe deer based on Raman spectroscopy and FTIR, which suggested that the experimental group had obvious spectra alteration corresponding to proteins, DNA, phospholipids, and glycogen. Although there are currently no reports on human testes, it is believed that the impact of adverse environmental factors on male reproductive health will be a meaningful new hotspot in the near future.

## 4 Application in Seminal Plasma Analysis

### 4.1 Assessment of Semen Quality

Semen is a mixture of sperm and seminal plasma, among which seminal plasma accounts for more than 95%. It is not only a necessary medium for sperm transport, but also contains substances necessary to maintain sperm life, which is also closely related to sperm function, as early evidence demonstrated that seminal plasma contains influence factors of male fertility (Diamandis et al., 1999). As a consequence, seminal plasma may contain important biochemical markers that can be measured non-invasively and reflect the quality of semen at the same time. Huang et al. (2011) found that the ratio of the Raman peak intensity at 1,449 cm^−1^ and 1,418 cm^−1^ assigned to tryptophane and lipid respectively in normal and abnormal semen samples was significantly different (*p* < 0.05). The sensitivity and specificity of diagnostic algorithm generated by combining PCA and LDA, were 73 and 82% respectively, and the area under the ROC curve was 0.823. Further research demonstrated that the normal and abnormal seminal plasma spectra obtained by different laser polarizations including non-polarized, linearly polarized, right-handed circular polarization and left-handed circular polarization were all different. As a result, the diagnostic algorithms developed based on this had different diagnostic capabilities when distinguishing normal and abnormal semen. Among them, the best diagnosis result was when the left-handed circularly polarized laser was used for excitation, and the sensitivity and specificity were 95.8 and 64.9% respectively. This might be due to the presence of a large number of chiral biomolecules in seminal plasma, just like phosphatase, aminopeptidase, glycosidase, hyaluronidase and mucin (Chen et al., 2012). In conclusion, the above studies demonstrated that Raman spectroscopy of seminal plasma can evaluate the quality of semen with high sensitivity and specificity.

### 4.2 Metabolomics Analysis of Seminal Plasma

Recent years have witnessed a spurt of progress in metabolomics, seminal plasma metabolomics research has also gradually attracted attention recently. Grounding on Raman spectroscopy, Gilany et al. (2018) constructed a seminal plasma metabolomics fingerprint for 20 patients diagnosed with NOA. Combined with the testicular biopsy results, they found that the metabolomics fingerprints of the two groups with positive sperm extraction (TESE (+)) and negative sperm extraction (TESE (−)) could be distinguished by PCA. What’s more, the signal intensity at 2,800–3,000 cm^−1^ assigned to the −CH group was stronger in TESE (−) patients than in TESE (+) patients, suggesting that TESE (−) patients were in a state of oxidative imbalance. In summary, the above results indicate that seminal plasma may have potential biomarkers for judging the presence of sperm in testicular tissues of NOA patients, and seminal plasma metabolomics detection based on Raman spectroscopy technology might be an ideal tool for non-invasive detection of spermatogenesis. Combining chemometric methods such as PCA and discriminant analysis, the team had further improved the classification model recently and found that although the semen analysis, medical history, and physical examination of idiopathic infertility men were normal, the classification model could still accurately distinguish the seminal plasma of idiopathic infertile men and normal fertile men which indicated that the microstructure of the seminal plasma metabolome of idiopathic infertility and fertile men was different (Pourasil and Gilany, 2021). Although it is still currently impossible to directly infer the cause of idiopathic infertility based on this, this study makes seeking the cause of the difference in seminal plasma metabolomics based on Raman spectroscopy possible.

## 5 Application in Sperm Analysis

Being the pioneer of the field of sperm analysis, Kubasek et al. (1986) first applied Raman spectroscopy to the study of salmon sperm DNA configuration in 1986. Unfortunately, in the following decades, the development and application of Raman spectroscopy in sperm analysis did not make significant progress. In recent years, with the update of equipment and the improvement of methodology, Raman spectroscopy to the scientific research and clinical application of sperm analysis have been applied in more and more researches.

### 5.1 Characterizing the Biochemical Information of Sperm

Whereas the characteristics of the chemical composition of different regions of sperm, combined with multivariate analysis methods, the three-dimensional Raman imaging of sperm cells could clearly show the nucleus located in the head, the mitochondria located in the middle and other organelles. Owing to single-point local Raman spectra could be obtained within hundreds of milliseconds, this technology may be applied for rapid fertility testing to examine the biochemical information and functional status of important organelles (such as mitochondria or nucleus) (Meister et al., 2010). Conducting a label-free imaging and biochemical characterization study on sperm cells, Amaral et al. (2018) found that different areas of sperm (head, middle and tail) could be clearly distinguished by Raman microscopy. However, there were no significant differences in the spectra of specific regions of sperm cells of four different species (humans, mice, Cynomolgus monkeys and sea urchins), which meant that the basic components of sperm were biochemically similar among species.

### 5.2 Identifying Normal Sperm

According to WHO reports, the infertility rate of couples of childbearing age has reached 15% globally, while half of the causes of infertility lie in men (Jungwirth et al., 2012). In this context, semen analysis is the most important auxiliary examination for men with infertility. In clinical practice, morphology and sperm motility are often seen as the main evaluation indicators to select normal sperm for assisted reproductive technology, while morphology and sperm motility alone are not enough to assess sperm fertilization ability. On account that we cannot evaluate sperm nuclear DNA integrity and chromatin function status through routine inspections that may affect sperm fertilization ability, these so-called normal sperm are not perfect. As a non-invasive, label-free, and emerging detection technology that can provide molecular biochemical fingerprint information, Raman spectroscopy has been demonstrated in more and more studies that it has great potential for identifying normal and capable sperm for fertilization.

#### 5.2.1 Identification Based on the Integrity of Sperm Nuclear DNA

In general, the sperm nuclear DNA (nDNA) damage model was induced by ultraviolet radiation (Mallidis et al., 2011; Da Costa et al., 2018), Da Costa et al. (2018) found that the peaks at 1,094 cm^−1^ and 1,050 cm^−1^ of the symmetrical stretching vibration of the PO_4_^-^ main chain of DNA could clearly distinguish the spectra of the control group and the experimental group. Sánchez et al. (2012) used Fenton reaction to induce the oxidative damage model of semen samples and obtained similar results. The study further used the intensity ratio of the Raman peak (1,050 cm^−1^/1,095 cm^−1^) to estimate the percentage of sperm with nDNA damage, and the performance was comparable to the most reliable flow cytometry in clinical practice. Both Raman spectroscopy and FTIR were used to double-verify the signal changes in the DNA backbone-related regions, which proved that the Raman signal changes in the 1,050–1,100 cm^−1^ region were meaningful, not accidental. Interestingly, Huang et al. (2014) found that the intensity ratio of 1,055 cm^−1^ to 1,095 cm^−1^ might be a potential biomarker for assessing sperm nDNA integrity. The above studies imply that the intensity ratio of 1,055 cm^−1^ or 1,050 cm^−1^ to 1,095 cm^−1^ may have practical application value in assessing the integrity of sperm nDNA. However, on account of the small sample size and the large heterogeneity of the experimental conditions, the results of other studies were also very different except for the two (Mallidis et al., 2011; Pereira et al., 2019). As a consequence, there is no sufficient evidence that the ratio of the two Raman shifts can be universally used to assess the integrity of sperm nDNA.

#### 5.2.2 Identification Based on Chromatin Function Status

Apart from the integrity of nDNA, the packaging efficiency of chromatin DNA and the condensed state of chromatin are also significant factors affecting sperm fertilization. Huser et al. (2009) provided evidence obtained from the analysis of single sperm, demonstrating that the DNA packaging in abnormally shaped sperm was different from that of normal-shaped sperm by Raman spectroscopy, abnormally shaped sperm might contain DNA that was not properly repackaged by protamine during sperm cell development. More importantly, they found that the properties and efficiency of DNA packaging in sperm considered to have normal morphology were quite different. As a consequence, if sperm is selected only by morphological indicators, a large part of them will contain improperly packaged chromatin DNA. Recently, Jahmani et al. (2021) used chromatin A3 (CMA3) staining results as the standard for chromatin aggregation levels and found that the median intensity of Raman peaks at 670 cm^−1^, 731 cm^−1^, 785 cm^−1^, 1,062 cm^−1^, 1,098 cm^−1^, 1,185 cm^−1^, 1,372 cm^−1^, 1,424 cm^−1^, 1,450 cm^−1^, 1,532 cm^−1^, 1,618 cm^−1^, and 1,673 cm^−1^ were significantly different between two groups of CMA3 ≤ 41% and CMA3 > 41%. Among them, the first seven peaks were assigned to the vibration of the DNA structure, and the last five peaks were assigned to the protein. Non-invasive confocal Raman spectroscopy makes an epoch in evaluating the level of sperm chromatin aggregation.

#### 5.2.3 Selection Based on the Combination of Zona Pellucida

Prophase studies have shown that the use of zona pellucida (ZP)-bound sperm for intracytoplasmic sperm injection (ICSI) urges higher embryo quality and implantation rate than conventional ICSI (Liu et al., 2011), so the research of a new method for sperm selection based on the binding of ZP is a very meaningful subject. Liu et al. (2013) used Raman microscopic spectroscopy to distinguish between ZP-bound sperm and unbound sperm. Compared with unbound sperm, in the acrosome region of ZP-bound sperm, two slightly low-intensity areas (800–900 cm^−1^ and 3,200–4,000 cm^−1^) associated with DNA and RNA backbones move to high-intensity levels. This may be related to the rupture of sperm cell membrane and outer acrosomal membrane after ZP-bound sperm acrosome reaction, leaving only a layer of acrosome outside the nucleus. In addition, this difference may also be caused by the different functional status of the nucleus (such as nDNA integrity).

### 5.3 Sorting of X and Y Chromosome Sperm

It’s worth mentioning that the sorting of sperm cells carrying X and Y chromosomes is very interesting, especially in animal production management systems and genetic improvement projects. Although the two types of cells contain the same amount of autosomal chromatin, the sperm with the X chromosome contains more total DNA than the sperm with the smaller Y chromosome (Li et al., 2016). Theoretically, in a certain volume, the total DNA concentration of the Y sperm cell is significantly lower than X sperm cell. Based on this, a method of Raman spectroscopy was proposed to effectively identify X- and Y-bovine sperm cells. The most obvious spectral difference between the two sperm cells appeared at the peaks at 726 cm^−1^, 785 cm^−1^, and 1,581 cm^−1^, and these peaks were the circular breathing patterns assigned to DNA bases (De Luca et al., 2014; Ferrara et al., 2015). Among them, the former study found that spectrum in the head and neck area could evaluate the biochemical differences between different sex chromosomes, and the accuracy of identification was as high as 90% (based on the results of flow cytometry). And the latter one used Raman spectroscopy combined with digital holography technology, making an epoch in completely unmarked sperm morphology selection and gender identification.

### 5.4 Other Applications

Apparently, the application of Raman spectroscopy in sperm research is far above that. Raman spectroscopy has been used as an auxiliary evaluation method in many studies to determine whether certain drugs and interventions can improve sperm quality (Li N. et al., 2014; Chen et al., 2014; Wu et al., 2015). Although these studies are in the exploratory stage, it is believed that with the advancement of technology, Raman spectroscopy may become a general auxiliary evaluation method in this field. In the past few years, many evidences have indicated that epigenetic regulation including sperm nDNA methylation may play a role in idiopathic male infertility (Gunes et al., 2016), D’Amico et al. (2020) applied resonance Raman spectroscopy to the study of genomic DNA methylation, indicating that simple and cheap Raman spectroscopy technology launched a new way for the screening of the cause of idiopathic diseases.

## 6 Discussion and Conclusion

To sum up, because of its characteristics of non-invasive, non-labeling and real-time *in situ* detection, the research and application of Raman spectroscopy and its derivative techniques in male reproductive system cover the molecular, cellular, tissue and organ levels, showing Raman’s unique advantages and great potential. The portable Raman spectrometer developed in recent years can be used for real-time *in vivo* and *in vitro* visual detection (Wang et al., 2015; Lane et al., 2018), compared with the Raman spectrometer for laboratory research, it is smaller and more convenient, and can realize rapid testing at bedside, during surgery, multi-scene and multi-purpose. No one doubts that these advantages will put it on the fast track of clinical transformation. If a relatively standard diagnostic algorithm is realized, this technique will have a good prospect in assisting prostate cancer surgery and micro-TESE.

Undeniably, there have been so many exciting studies that can achieve the Raman detection of the subcellular structure of single sperm. Unfortunately, the research on living sperm or sperm population is still facing many challenges, most of the existing reports are the study of semen smears, so that they can’t be further used in the treatment of ART. To make it, at least three conditions must be met: one is to maintain sperm activity, the other is to keep the sperm relatively fixed, and the third is to acquire the signal in a liquid environment. de Wagenaar et al. (2015) cleverly designed a microfluidic device to capture live sperm for single sperm analysis. However, analysis techniques relying on fluorescent labels wound damage sperm quality. On the contrary, non-invasive, label-free Raman spectroscopy technology may be a promising alternative technology, while current research has not yet demonstrated how effective it is. In order to detect the Raman signal of live sperm, Da Costa et al. (2018) used poly-lysine slides to fix the sperm in a liquid environment. However, the Raman spectrum difference of DNA damage caused by ultraviolet radiation could only be observed under dry conditions, but not in aqueous medium, the mechanism of which is not yet clear so that more experimental studies are needed to be obtained for the reason of the decreased ability of Raman spectroscopy to detect sperm DNA changes in the hydrated state. Obviously, there is still a long way to go for evaluating the quality of sperm based on Raman spectroscopy and applying it on subsequent treatment.

In addition, there are several problems worthy of our consideration in the application of Raman spectroscopy and its derivative techniques in the male reproductive system. The first is the safety of Raman spectroscopy, especially when it is used to evaluate normal sperm and for follow-up ART therapy. Although this technique is widely considered to be non-invasive, considering the damage of ultraviolet radiation to sperm nDNA (Mallidis et al., 2011; Da Costa et al., 2018), we can’t guarantee whether laser has adverse effects on sperm genetic material during Raman test. If sperm with genetic material defects caused by the Raman test are used in ART, it may lead to adverse pregnancy outcomes such as miscarriage and congenital malformations, which is ethically unacceptable. Secondly, a large number of Raman peaks can be obtained from prostate, testicular tissue and semen samples with few known peak attributions, so more research is needed to further explore and improve the Raman peak information database. Another challenge is that the results of Raman spectra are easily disturbed by external factors, which leads to the heterogeneity of different studies. No matter the room brightness, temperature, laser wavelength, intensity, exposure time and other equipment parameters of the test environment may affect the test results, resulting in poor unity between different studies, so it is difficult to establish the standard of Raman spectroscopy in practical application, which greatly limits its application in reproductive andrology and even other clinical disciplines.

In summary, Raman spectroscopy has emerged in the research and application of the male reproductive system, which can scan organs, tissues, cells and body fluids in real time without damage and labels, and expose subtle biochemical changes. It has become a potentially useful tool for disease diagnosis, searching for potential biomarkers and sorting normal sperm. And the development of portable Raman spectrometers and corresponding diagnostic algorithms will greatly accelerate the clinical transformation of Raman spectra. However, a lot of work is still needed to strengthen the standardization and safety evaluation of this technology. It is believed that Raman spectroscopy will open up a new situation in male reproductive medicine in the future.
